# Tunable Coupler–Augmented Microrings: Reconfigurable Q‐Factor Control for Foundry‐Scale Quantum Light Sources

**DOI:** 10.1002/nap2.70043

**Published:** 2026-03-20

**Authors:** Jan Heine, Juan Samuel Sebastián Durán Gómez, Michael Kues

**Affiliations:** ^1^ Institute of Photonics (IOP) Leibniz University Hannover Hannover Germany; ^2^ Hannover Centre for Optical Technologies Leibniz University Hannover Hannover Germany; ^3^ Cluster of Excellence PhoenixD Leibniz University Hannover Germany

**Keywords:** integrated quantum photonics, quantum light sources, tunable cavities

## Abstract

High‐brightness–integrated quantum light sources represent a fundamental element for large‐scale photonic quantum technologies. Integrated microring resonator (MRR) in highly nonlinear and ultralow‐loss material platforms enable entangled photon‐pair generation, but device performance is affected by fabrication tolerances and can thus not be judiciously controlled. Redundant designs based on parameter variation are impractical for chip‐scale circuits, as the sheer number of parameters would be too complex. To address this, passive photonic circuits must be combined with active control elements to optimize photon‐pair emission and to match emission characteristics among sources. Here, we present a comprehensive study of an actively tunable, interferometrically coupled integrated MRR implemented in silicon nitride (SiN). By manipulating two thermally controlled phase shifters, the ring–bus coupling and the resonator phase can be dynamically adjusted, enabling post‐fabrication control of its quality factor (Q‐factor) between 10^5^ and 10^6^ to optimize the photon emission bandwidth and the pair generation rate. We directly demonstrate for the first time that the optimal coupling and emission characteristics do not occur at critical coupling and vary across frequency modes due to dispersion effects, in good agreement with our theoretical model. These findings highlight the role of adaptive integrated photon sources for single‐batch fabrication of reconfigurable devices with application‐specific spectral properties.

## Graphical abstract

Scalable quantum photonics require robust realization of quantum light sources for foundry‐scale fabrication. An interferometrically coupled silicon nitride microring resonator enables post‐fabrication control of bus–ring coupling. Thermal phase shifters adjust the Q‐factor and the pair generation rate, revealing that optimal generation does not occur at critical coupling and that emission characteristics are dispersion‐dependent. This approach enables single‐batch fabrication of quantum light sources with reconfigurable Q‐factors.

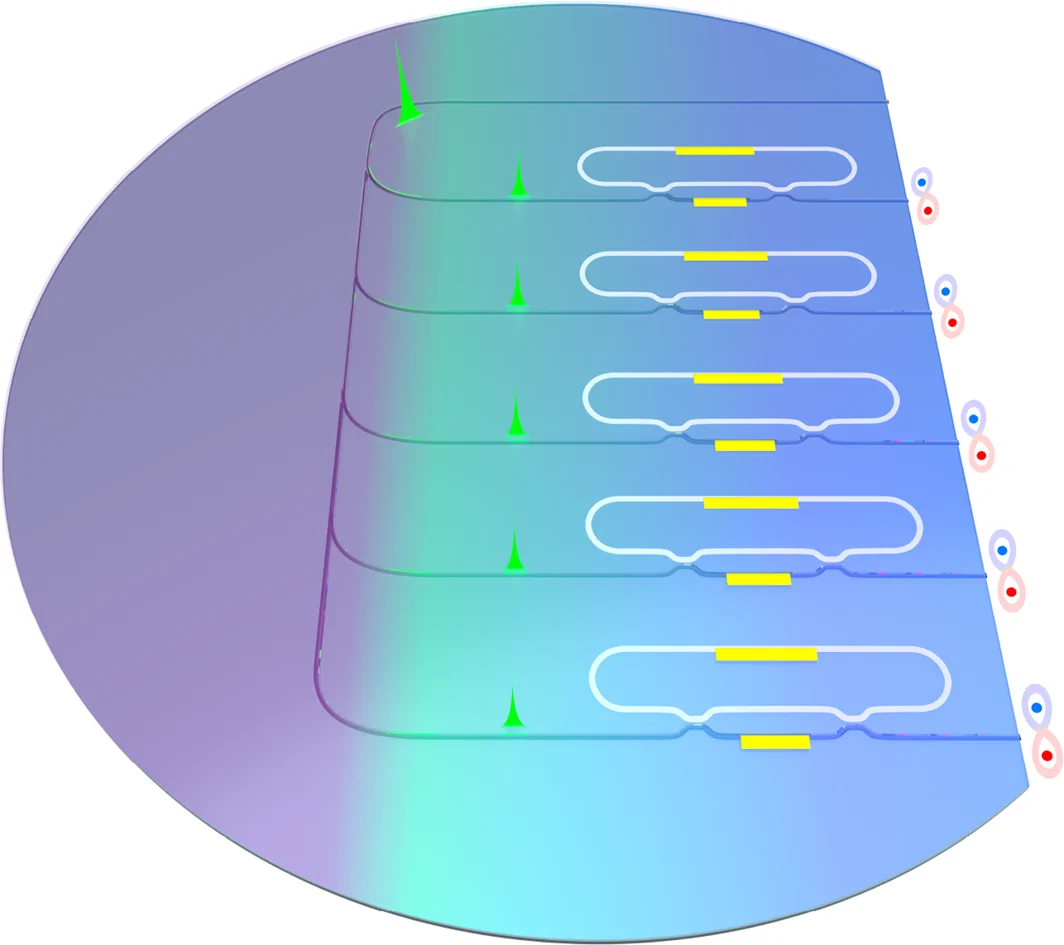

## 1 Introduction

Entangled photon‐pair sources are essential components in quantum photonic technologies, enabling secure quantum communication [1, 2, 3], photonic quantum computing [4, 5, 6], and advanced quantum sensing [7, 8]. Particularly, entanglement‐based quantum key distribution (QKD) protocols and quantum computing need to be built upon high brightness sources with emission in the 1550‐nm telecom band [2, 9]. Sources based on spontaneous parametric down‐conversion (SPDC) (second‐order $\chi^{\left(2\right)}$) and spontaneous four‐wave mixing (SFWM) (third‐order $\chi^{\left(3\right)}$) have emerged as a driver for both lab‐scale experiments as well as industrial implementations in quantum key distribution and quantum computing efforts [10, 11]. Microring resonators (MRR) in material platforms including silicon on insulator (SOI) [12, 13], fused silica (SiO_2_) [14], aluminum gallium arsenide (AlGaAs) [2, 15], SiN [16, 17], and others evolved as an advantageous concept for generating entangled photon states on chips, particularly via SFWM in high‐quality factor and low‐loss structures [18, 19]. Combined with dispersion engineering, MRR can provide narrowband entangled photons spanning many frequency modes, which are fundamental for high‐dimensional quantum states and quantum optical coherence tomography [16, 19, 20]. The narrow linewidths supported by MRR together with engineered waveguide dispersion properties allow precise spectral control [21], for example, for direct interfacing with quantum memories (solid state systems based on erbium ions) and wavelength‐division multiplexing schemes. Among all integrated photonic platforms, SiN has been established as a platform with low loss [17], which is an important requirement for enabling integrated cavities. SiN has been widely explored for applications ranging from visible [22, 23, 24] to mid‐infrared wavelengths [25] in classical applications, as well as the generation of quantum frequency combs [26, 27]. Higher confinement than, for example, SiO_2_ enables small device footprints, whereas low loss and compatibility with CMOS fabrication boost photon pair generation rates through strong resonant enhancement [12, 28] and enable large‐scale photonic circuits with volume manufacturing capabilities, respectively. This unique combination of device and material platform positions integrated MRR in SiN as a scalable, industry‐ready solution for entangled photon pair sources [14, 29, 30, 31]. However, high‐performance photon pair sources based on MRR employ fixed bus–ring coupling regions with a required careful choice of the ring–bus coupling (e.g., to maximize intracavity field enhancement or to match the resonator linewidth to another optical mode), limiting post‐fabrication control and making performance highly sensitive to fabrication variations and changes in operating wavelength. This tolerance to fabrication variations such as geometric errors (waveguide width, height, and gap variations) or material transmission could only be achieved by fabricating parameter variations of the same design on a wafer, which becomes infeasible as circuit complexity increases. A related approach was demonstrated by Liu et al. using a dual‐interferometer–coupled silicon MRR, where independent tuning of the pump, signal, and idler resonances enhanced photon‐pair spectral purity [32]. Although interferometric phase shifters enable post‐fabrication tuning of the effective bus–ring coupling, in an asymmetric Mach–Zehnder interferometer (MZI), their wavelength‐dependent response links resonance shifts and coupling strength, limiting broadband control and systematic access to different coupling regimes over several MRR resonances, often requiring repeated thermal tunings of mode pairs. In this work, we employ an MRR with a tunable MZI coupler, enabling dynamic control over the bus–ring coupling coefficient after fabrication. In addition, we experimentally study for the first time the impact of dispersion on the optimal coupling. This tunability enables reconfiguration of the MRR between the overcoupled, critically coupled, and undercoupled regimes to tailor photon‐pair brightness, overall source emission bandwidth, and single‐photon bandwidth. The inclusion of tuneable couplers relaxes lithographic precision requirements in the coupling region, directly improving wafer‐scale yield. Devices that would otherwise be discarded due to coupling mismatch can be brought into specification through post‐fabrication tuning, reducing cost, and accelerating prototyping cycles. This reconfigurability not only enhances the functional versatility of the device but enables high‐yield, scalable, and application‐agnostic building blocks for quantum photonics.

## 2 Device Design and Experimental Characterization

An SiN photonic integrated circuit (PIC), fabricated in an 800 nm silicon nitride layer height multi‐project wafer run by Ligentec SA was used for this study. An all‐pass racetrack ring resonator with a cavity length *L* = 5721 μm giving a free spectral range (FSR) of 25 GHz was formed from a ridge waveguide–SiO_2_ cladding with a cross‐section of 1500 nm width and 800 nm height, which provides slightly anomalous dispersion of 67.8 ps nm^−1^ km^−1^ in the transverse electric (TE) mode. A tunable coupler was designed with two 11% s‐bend–shaped directional couplers (using 1 μm wide waveguides to shorten the coupling length) forming a Mach–Zehnder interferometer (Figure 1a). A thermal phase shifter (PS_1_) in one arm of the interferometer was employed to control the effective coupling. The racetrack resonator phase was controlled with a second thermal phase shifter (PS_2_). The directional coupler, the key component controlling the effective Q‐factor, remains robust to realistic fabrication variations: height variations of ± 10 nm and width or gap variations of ± 50 nm result in coupling coefficients between 5% and 23%, which can be compensated via thermal phase tuning of PS_1_ in the MZI. Light was coupled onto and off the chip using mode converters compatible with standard single‐mode telecom fiber, exhibiting a coupling loss below 1.5 dB per facet for the TE polarization. The measured total fiber–chip–fiber insertion loss was 3.27 dB. All measurements in this work were done in the TE mode, due to the lower coupling loss. The chip was assembled and sealed in a butterfly package with a Peltier and temperature sensor for precise temperature stabilization, equipped with a fiber array for optical access and electrically connected by wire bonding. This fully packaged module greatly enhanced the stability and repeatable tunability of the device.

**FIGURE 1:**
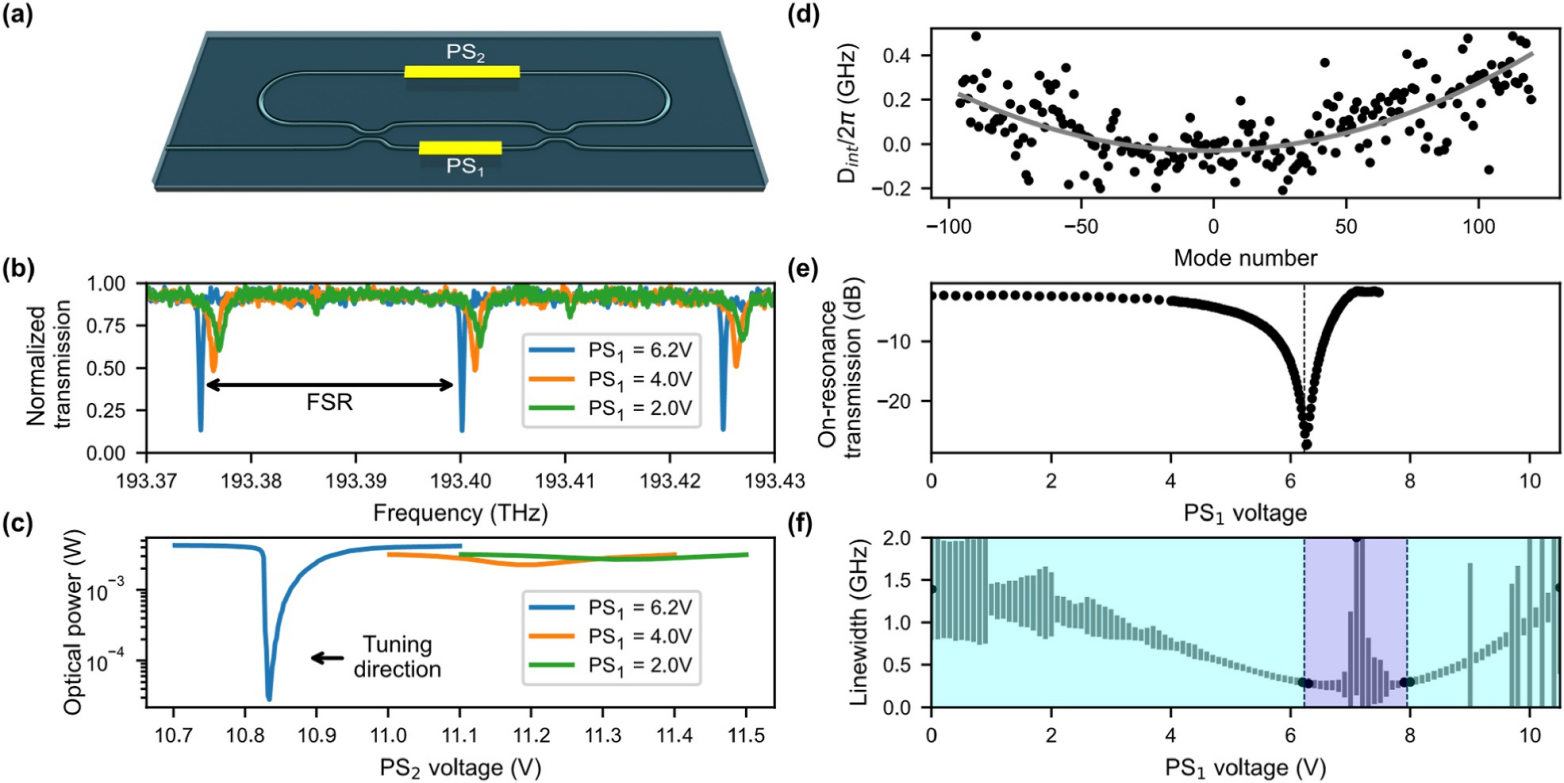
Characterization of the all‐pass ring resonator with a tunable coupler. (a) A schematic render of the racetrack resonator design, indicating the positions of the thermal phase shifters PS_1_ (coupling) and PS_2_ (resonator phase). (b) Spectra for different coupling regimes, from near‐critical to strongly overcoupled for three resonances around the pump. (c) Optical power traces for fixed PS_1_ settings, with varying PS_2_. The coupling shifts, as PS_1_ changes the resonator phase. (d) Integrated dispersion $D_{int}(\mu )$, obtained by fitting the resonance frequencies from (a). (e) On‐resonance transmission measured for different PS_1_ voltages using the data in (c). The minimum at 6.260 V corresponds to critical coupling (dashed line). The asymmetry is a result of the most quadratic dependence of phase shifter voltage on heater power. (f) Resonator linewidth for different PS_1_ voltages, calculated from the spectra in (b). Gray bars indicate the fitting error, and dashed lines mark critical coupling for two different voltages. Note that the linewidth estimation is error prone for the strongly overcoupled (cyan region) and the undercoupled (purple region) regime, due to the low extinction in both regimes.

We characterize the resonator and its tunable coupler, with the help of a narrow‐linewidth continuous‐wave (CW) laser (center frequency = 193.400 THz and linewidth *δν*_*p*_ = 10 Hz) and the two on‐chip phase shifters PS_1_ and PS_2_ (Figure 1b,c). The on‐chip power used for all photon‐pair generation experiments was estimated to be 9.6 mW, limited by the insertion loss and the optical power limit of the fiber components. To determine the FSR and integrated dispersion of the ring resonator (see Figure 1d), optical spectra were measured with a high‐resolution optical spectrometer (180 MHz typical resolution bandwidth). The resulting spectra, normalized to the off‐resonance power at 193.39 THz, are shown in Figure 1b for the three different applied voltages. To measure the extinction of the resonances for different voltages applied to the phase shifter PS_1_ and to determine the voltage setting for critical coupling, the ring phase shifter PS_2_ was adjusted to find the minimum of the laser light transmissions for different settings of PS_1_ (Figure 1e). Recording the optical power transmitted through the device, the on‐resonance extinction can be determined. The off‐resonance transmitted power was manually determined to be 5.40(3) mW. Voltage tuning of PS_2_ is shown in Figure 1c for different PS_1_ voltages. We note that the tuning direction is paramount for achieving stable coupling, when the ring is close to critical coupling. Further, the transmission minimum is close to the estimated dynamic range of the used power meter of 32 dB for the chosen laser input power, which marks the lower limit for this measurement. The minimum can nonetheless be clearly identified and used to determine the critical coupling voltage PS_1,crit_ = 6.260(5) V. The average resonance linewidth is determined by fitting the measured spectra with an idealized resonator spectrum $P\left(f\right)=\Sigma_{n}^{-20\leq n\leq 20}L\left(f0+n\times FSR+D_{\mathrm{int}}\left(n\right)-f_{\text{pump}}\right),$ where $L\left(f\right)=A/\left(1+4\frac{f^{2}}{\gamma^{2}}\right)+B$ describes individual Lorentzian resonances with γ as the full width at half maximum (FWHM) (Figure 1f) and A and B as on‐resonance extinction and constant offset for the fit. The resonances, approximated by the idealized resonator function, were individually fit with a Lorentzian function, using the approximated parameters as starting points to accurately determine the resonance bandwidths. The analysis demonstrates the tunability of the MRR linewidth between 1.389(597) GHz and 0.254(71) GHz, where the error is one standard deviation, obtained from the fitted resonances.

### 2.1 Ring–Bus Coupling Effect on Photon Pair Generation

To experimentally validate the performance of the proposed tunable MRR, we characterized its photon‐pair emission properties under controlled coupling conditions. For emission characterization, a narrow linewidth laser was filtered with a high‐isolation DWDM filter and coupled to the PIC (see Figure 2). The coupling was tuned in steps with voltage adjustments of PS_1_, whereas the ring phase was adjusted with PS_2_ so that one resonance coincided spectrally with the laser at 193.400 THz. Photon pairs and residual laser light were separated by another DWDM filter after outcoupling from the PIC. Signal and idler photons were spectrally filtered and routed to two SNSPDs (∼12 ps jitter) for coincidence counting. The laser power measurement was used both to align the ring resonance with the laser frequency and to monitor resonator extinction. Owing to the MRR's narrow linewidth, the high temporal resolution of the time‐to‐digital converter (∼2.8 ps) and the SNSPDs (∼12 ps), the cross‐correlation resolves the spectral properties of the signal and idler resonances and allows estimation of the Q‐factor of the modes by fitting an exponential decay function. Coincidences were counted within the FWHM of the correlation histogram, and accidentals were subtracted using the fitted noise floor to approximate the true coincidences. The coincidence to accidental ratio (CAR) was calculated as $\text{CAR}=\frac{CC_{\text{true}}}{\text{AC}}$, where AC is the accidental counts in the same window as the coincidences.

**FIGURE 2:**
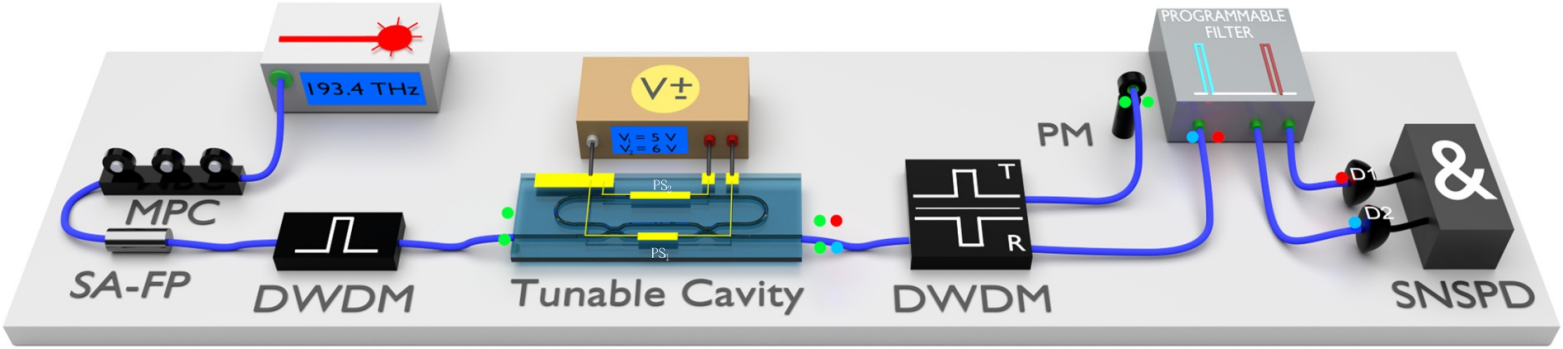
Schematic representation of the experimental setup for the reconfigurable photon source. A CW laser is firstly polarization aligned with the slow axis polarization on the PIC and filtered before being coupled to the chip. A manual polarization controller (MPC) in combination with a slow‐axis fiber polarizer (SA‐FP) aligns the laser to excite TE polarization in the chip, followed by filtering with a 100 GHz C34‐dense wavelength division multiplexing (DWDM) with high‐channel isolation. The thermal phase shifters are controlled with a programmable voltage source (resolution < 1 mV) to adjust the coupling phase and the ring resonator phase with PS_1_ and PS_2_, respectively. Photon pairs produced are out‐coupled and filtered with a 200‐GHz C34‐DWDM to separate the correlated photons from the pump. The transmission port of the DWDM is connected to a power meter (PM) to spectrally align the laser frequency with the MRR resonance and to monitor the stability of the coupling, whereas the reflected port is sent to a programmable filter to split signal and idler photons, which afterward are send to two superconducting nanowire single‐photon detectors (SNSPDs) to correlate them in time.

The datasets for correlated modes 20, 30, 70, and 90 (i.e., signal photons at μ=20 and idler photons at μ=−20 etc.) are shown in Figure 3a as a function of the voltage applied to PS_1_. The first two mode pairs, which are close to the excitation frequency, have considerably less dispersion than the other two mode pairs, allowing for a clear comparison between the two regimes. The shown Q‐factor for each pair is calculated from the cross‐correlation histogram as the average of the two resonances, and the Q‐factors are very similar for all mode pairs considered. To better understand the dependence of the pair generation rate (PGR) on the coupling and dispersion, we estimated the number of correlated photon pairs as a function of the mode index μ and the loaded Q‐factor $Q_{l}$. By considering the dispersion‐induced resonance shift and the field enhancement of the signal and idler modes, the correlated photon‐pair rate can be expressed as (see Appendix A for details)

(1) $$N_{c,\mu }={\mathrm{AC}}_{-\mu }C_{\mu }\frac{\pi \;\left(\omega_{\mu }+\omega_{-\mu }\right)}{8Q_{l}}\frac{1}{1+\frac{4Q_{l}^{2}{\left(D_{2}\mu \right)}^{2}}{{\left(\omega_{\mu }+\omega_{-\mu }\right)}^{2}}},$$

where A, $C_{\pm \mu }$, and $D_{2}$ are parameters related to the nonlinear conversion efficiency, field enhancement, and dispersion, respectively. The detected coincidence rate shows a good agreement with the dispersion‐less approximation of Equation (1) for mode 20. The maximum value corresponds to a loaded Q‐factor of $Q_{l,\mu =20}=0.855Q_{l,\text{crit}}\approx \frac{6}{7}Q_{l,\text{crit}}$ as the theory predicts. We determined a maximum on‐chip PGR of 334 kHz, after correcting for optical losses and detector efficiencies ($\text{PGR}=\frac{\text{CC}}{\eta_{s}\eta_{i}}$, with $\eta_{s}=0.0905$ and $\eta_{i}=0.0967$). For a larger dispersion, the optimum detected photon pair rate shifts toward stronger overcoupling. The lower overall coincidence count rate is in part a result of the phase mismatch of the nonlinear process due to dispersion. The maximum coincidence rate was estimated by numerically optimizing the Equation (1) for different coupling conditions (indicated as vertical color lines in Figure 3a), where the Q‐factor of each resonant mode was expressed relative to the experimentally measured critical Q‐factor. We observe generally good agreement between the estimated and measured maximum coincidence rates for the four modes considered. The trend of the CAR versus coupling remains similar regardless of dispersion, showing a plateau in the CAR within the overcoupled regime for all measured modes. We suspect the absolute value is set by detector dark counts and nonresonant photon‐pair generation in the PIC. These measurements provide practical design insight for MRR on platforms with a significant integrated dispersion, and they highlight the advantages of the tunable coupler MRR for an integrated entangled photon‐pair source. By adjusting the coupler, one can reduce the photon‐pair detection rate to achieve a > 2× increase in CAR when a higher signal‐to‐noise ratio is required.

**FIGURE 3:**
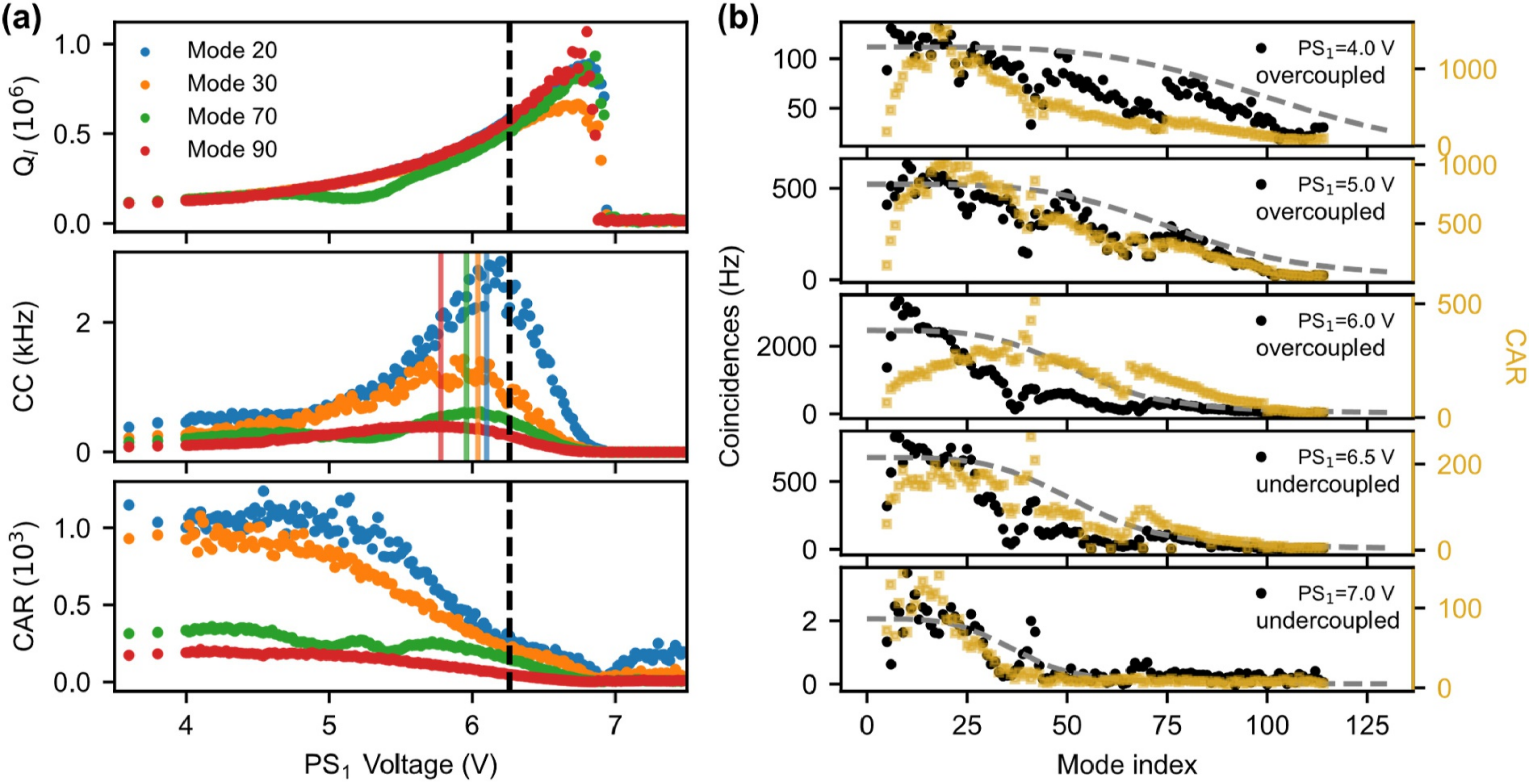
(a) Q‐factor, coincidence counts, and CAR as a function of the voltage applied to PS_1_ for four modes, two with low (modes 20 and 30), and two with high‐integrated dispersion (modes 70 and 90). The dashed line denotes the voltage for critical coupling, and the vertical continuous lines indicate the predicted maxima positions of the coincidence rate, according to a numerical optimization of Equation (1), in good agreement with the measured data. Frequency mode 20 shows excellent agreement with the dispersion‐less theory, whereas the modes with higher dispersion show that more overcoupled resonances are favorable for a high–photon‐pair detection rate. (b) Measured main diagonal of the joint spectral intensity (JSI) for different resonator–bus couplings, set by PS_1_. The simulated conversion efficiencies, normalized to the measured counts, for the corresponding Q‐factors are shown in gray. The deviation of simulation and experiment close to the critical coupling (PS_1_ = 6 and 6.5 V) is most likely attributable to the use of the theoretical‐integrated dispersion from the model, without accounting for the exact resonance positions.

### 2.2 Coupling Dependence on the Joint Spectral Intensity

An essential parameter for the practical implementation of photon‐pair sources is the photon‐pair generation bandwidth, beacuse it directly determines the range of frequencies, useful for, for example, quantum information processing or QKD [2, 27]. We analyze the effect of the ring‐to‐bus coupling on the MRR photon pair source by measuring the main diagonal of the JSI for selected PS_1_ voltages in all coupling regimes, to show the impact of the coupling on the JSI bandwidth and CAR. To make a meaningful comparison between experiment and theory, we simulate the JSI envelope, based on the measured Q‐factor of the MRR at the different coupling voltages. This was done by calculating the normalized main diagonal of the JSI (Equation 2), accounting both the cavity‐induced reduction of spectral overlap among pump, signal, and idler resonances and the waveguide dispersion obtained from effective index simulations. The result is adjusted to fit the measured count rate of the first 10 modes of the experimental JSIs. The normalized main diagonal of the JSI for a bi‐photon quantum state generated in an integrated cavity can be expressed as [16, 31, 33]

(2) $$|G_{\text{cav}}(\mu ){|}^{2}=\zeta_{0}\int_{-\infty }^{\infty }d\omega_{p}\;\;\alpha \left(\omega_{p}\right)\times \int_{-\infty }^{\infty }d\omega A_{\mu }^{2}(\omega )A_{-\mu \;}^{2}\left(2\omega_{p}-\omega \right){\mathrm{sinc}}^{2}\left(\frac{L\Delta \beta }{2}\right),$$

where *ζ*_0_ is a normalization parameter. The term $\Delta \beta =2\beta (\omega_{p})-\beta (\omega )-\beta (2\omega_{p}-\omega )$ corresponds to the phase‐matching condition, with *β* denoting the propagation constant. The function $\alpha (\omega_{p})$ represents the pump envelope, whereas $A_{\mu }^{2}\left(\omega \right)$ is the Airy function that characterizes the spectral response of the cavity in an all‐pass ring resonator [14] as follows:

(3) $$A_{\mu }^{2}\left(\omega_{\mu }\right)={\left|\frac{t_{\mu }}{1-r_{\mu }e^{i\beta_{\mu }\left(\omega_{\mu }\right)L}}\right|}^{2},$$

where $t_{\mu }$ and $r_{\mu }$ denote the transmissivity and reflectivity amplitudes of the cavity, respectively. The Q‐factor is linked to the reflectivity through $|r_{\mu }|=1-\frac{m\pi }{Q_{l}}$. We estimated the loaded Q‐factor for the different voltages of PS_1_ for our simulations. Importantly, the Airy function naturally leads to a frequency comb structure, where the resonance maxima appear at frequencies that satisfy the condition βL=2πm, with m representing the azimuthal mode index. The pump envelope was considered as a Gaussian function with a narrow linewidth of *δν*_*p*_ = 10 Hz. Solving Equation (2) for the different resonator Q‐factors allows us to compare the theoretical joint spectrum with the measured JSI. This comparison enables us to demonstrate that the tunable coupler does not negatively affect the ring resonator's performance. The generation bandwidth was investigated by performing cross‐correlation measurements of the frequency‐correlated modes, where a programmable spectral filter was used to separate signal and idler photons (see Figure 2). They were filtered with 20 GHz bandwidth, and coincidence histograms were recorded for all 110 MRR modes within the filter's bandwidth. This corresponds to a quantum frequency comb (QFC) spanning 5.5 THz. Figure 3b illustrates the main diagonal of the JSI for five different voltages of PS_1_, that is, different Q‐factors, showing overcoupled, close‐to critically coupled, and undercoupled pair generation rates and CAR. It can be observed that the generation bandwidth can be optimized by operating the device in the overcoupled regime, where the highest detected coincidence rates are obtained when the ring's Q‐factor approaches 85.7% of $Q_{l,\text{crit}}$.

We generally observe a good agreement between the estimated JSI and the measurements, with the notable exception of mode 30 shown in Figure 3b, for which a clear decrease in coincidence counts is observed. We attribute this discrepancy to the overlap between the ring transfer function and the MZI transmission: for a nonzero phase shift in one MZI arm, the entire interferometer becomes imbalanced, leading to a spectrally dependent transmission that can modify the apparent Q‐factor values.

The lowest overall performance is observed in the undercoupled regime, where the bandwidth is most limited and the coincidence rates decrease significantly due to the narrow linewidth and the low intracavity power enhancement resulting from poor extinction. By varying PS_1_, it is possible to optimize generation bandwidth at the cost of photon rate, CAR, generation rate, or photon linewidth depending on the application requirements.

Table 1 summarizes a benchmark comparison of representative integrated microring‐based photon‐pair sources reported in the literature, highlighting key performance metrics such as free spectral range, Q‐factor, on‐chip pair generation rate, and coincidence‐to‐accidental ratio. Compared to previous demonstrations, our device uniquely combines a high and tunable Q‐factor with a moderate FSR, as well as high PGR and CAR, enabled by post‐fabrication reconfigurable coupling.

**TABLE 1:** Benchmark comparison of key parameters and features of integrated MRR photon‐pair sources.


Platform	FSR (GHz)	Q‐factor	On‐chip PGR (kHz)	CAR	Relevant aspects	Reference
SiN + InP	199	9.67 × 10^4^	8.2(@3.9 mW)	80	Fully integrated hybrid source	[26]
Si	400	1.5 × 10^4^	1147(@61 μW)	81	Spectral purity focus	[32]
Si	∼ 2460	9.6 × 10^4^	83(@79 μW)	65	Low‐power pair generation	[34]
LiNbO_3_	137	1.28 × 10^5^	490(@12.3 μW)	1235	Modal efficient SPDC	[35]
SiN	25	10^5^–10^6^	334(@9.6 mW)	100–1000	Reconfigurable coupling	This work

## 3 Discussion and Conclusion

In conclusion, we have demonstrated a racetrack MRR with an interferometric coupler to vary the bus–ring coupling coefficient of the MRR. We characterize the device and demonstrate the tunability of the coupling and excellent suppression when critically coupled. The control over the bus‐to‐ring coupling allows for the first time to study the impact of the coupling coefficient on photon pair generation inside an MRR in the same device, removing variations in length, dispersion, loss, sidewall roughness, material, and layer height, which otherwise limit comparability. We demonstrate that the device remains tolerant to realistic fabrication variations, as coupling values can be compensated through thermal phase tuning in the symmetric MZI. We derive a theoretical model to predict the optimum coupling required to maximize the photon pair detection rate under the influence of dispersion. Compared to the zero‐dispersion case, dispersion leads to a shift of the MRR resonances with frequency, which causes a limited spectral overlap of pump‐, signal‐, and idler‐resonances. The result is that the coupling for which a maximum photon pair detection rate can be found shifts toward the overcoupled regime for increasing dispersion, well visible in our measurement. The experimental performance of the device demonstrates the applicability of tunable coupling for high‐performance photon‐pair sources. This approach reduces the need for costly design iterations or large‐scale parameter variations, as the coupling can be adjusted simply in experiments to optimize source bandwidth, generation rate, or CAR. As a result, the overall PIC design becomes more robust to fabrication variations that affect either the coupling coefficient or the MRR roundtrip loss. The obtained results can be directly applied to fixed coupler designs to optimize MRR photon‐pair sources based on highly dispersive materials for optimal bandwidth, CAR, or detected photon‐pair rate.

## Author Contributions

Jan Heine conceived and designed the experiment. Experiments were conducted by Jan Heine. The data were analyzed by Juan Samuel Sebastián Durán Gómez and Jan Heine, with input from Michael Kues. Juan Samuel Sebastián Durán Gómez performed the theoretical bandwidth simulations. The initial draft of the manuscript was written by Jan Heine and Juan Samuel Sebastián Durán Gómez. All authors contributed to the preparation of the final manuscript. The project was supervised by Michael Kues.

## Funding

This work received funding from German Federal Ministry of Research, Technology and Space (Bundesministerium für Forschung Technology und Raumfahrt; BMFTR), within the project InQL, the European Research Council (ERC) under the European Union's Horizon 2020 research and innovation programme under grant agreement No. 947603 (QFreC project), and the German Research Foundation (Deutsche Forschungsgemeinschaft; DFG) within the cluster of excellence PhoenixD (EXC 2122, Project ID 390833453).

## Conflicts of Interest

The authors declare no conflicts of interest.

## Data Availability

The data that support the findings of this study are available from the corresponding author upon reasonable request.

## Appendix A

### Theoretical Analysis of Ring Coupling for Photon‐Pair Emission

In the following section, we outline a model to calculate the spectral characteristics of photon‐pair generation in a single all‐pass MRR via SFWM, where the ring to bus coupling is a tunable parameter and the ring resonator performance is affected by dispersion.

The loaded Q‐factor is defined as $Q_{l}=\frac{\omega_{\text{res}}}{\Delta \omega }={\left(\frac{1}{Q_{i}}+\frac{1}{Q_{e}}\right)}^{-1},$ where $\omega_{res}$ and Δω are the resonance frequency and resonator FWHM linewidth, respectively. It can be decomposed into an internal $Q_{i}$ and external $Q_{e}$, describing the roundtrip loss and the coupling of the resonator to the bus, respectively. The intracavity pump power can be calculated from $P_{\text{cav}}=P_{p}*|F(\omega_{p}){|}^{2}$, with $\omega_{p}$ and $P_{p}$ being the pump laser angular frequency and on‐chip power, respectively, and $|F(\omega ){|}^{2}$ being the field enhancement factor [12], as follows:

(A1)
$${\left|F\left(\omega \right)\right|}^{2}=\frac{4v_{g}Q_{l}^{2}}{L\omega Q_{e}}\;\frac{1}{1+4Q_{l}^{2}\frac{{\left(\omega -\omega_{res}\right)}^{2}}{\omega_{res}^{2}}}.$$

On resonance, the enhancement has a maximum of

(A2)
$${\left|F\left(\omega =\omega_{\text{res}}\right)\right|}_{\max}^{2}=\frac{4v_{g}Q_{l}^{2}}{L\omega_{\text{res}}Q_{e}}\;.$$

To describe the dispersion, we obtain the effective refractive index for the TE_00_ mode using a mode solver. We considered a ridge waveguide with a width of 1500 nm and a thickness of 800 nm surrounded by SiO_2_. We can express the dispersion as the integrated dispersion, with the excitation mode ($\mu_{\omega_{p}}=0$) as the reference using the following equation [36, 37]:

(A3)
$$\omega_{\mu }=\omega_{0}+D_{1}\mu +D_{\mathrm{int}}\left(\mu \right),$$

where μ is the mode index and the term $D_{\mathrm{int}}\left(µ\right)\approx \frac{1}{2}D_{2}\mu^{2}+\text{…}$ can be expanded to account for dispersion of different order. Here, we expand only up to the second order, which is usually dominant for dispersive waveguides. Higher orders can be treated in the same way. In this formulation, $D_{1}⁄(2\pi )$ is equal to the FSR, $D_{2}$ is related to the second‐order dispersion D (respective $\beta_{2}$), and $\omega_{0}$ is the reference mode (pump mode).

The phase mismatch for a signal/idler mode becomes

(A4)
$$\Delta \omega \left(\mu \right)\approx -D_{2}\mu^{2}\;.$$

The pair generation rate into a single (signal/idler) mode is described as in [12] (with the ring resonator length L=2πR)

(A5)
$$N_{c,s⁄i}=A\int |F\left(\omega_{\text{res}}-\Delta \omega \right){|}^{2}\;|F\left(\omega_{\text{res}}+\Delta \omega \right){|}^{2}\;d\Delta \omega ,$$

with $A={\left(\gamma P_{\text{cav}}L\right)}^{2}$, depending on the intracavity pump power, MRR material, and geometry.

The dispersion introduces a shift in the maxima of the Lorentzian enhancement factor $|F(\omega ){|}^{2}.$ Considering Δω(μ), the equation becomes

(A6)
$$N_{c,s/i}=AC_{-\mu }C_{\mu }\int_{-\infty }^{\infty }\frac{1}{1+\frac{4Q_{l}^{2}}{\omega_{-\mu }^{2}}{\left(\omega -\frac{D_{2}\mu^{2}}{2}\right)}^{2}}\;\frac{1}{1+\frac{4Q_{l}^{2}}{\omega^{2}}{\left(\omega +\frac{D_{2}\mu^{2}}{2}\right)}^{2}}\;d\omega ,$$

with $C_{\mu }=\frac{4v_{g}Q_{l}^{2}}{L\omega_{\mu }Q_{e}}$, where we approximate $Q_{\mu }=Q_{-\mu }=Q_{0}=Q_{l}.$ This integral evaluates exactly to

(A7)
$$N_{c,\mu }=AC_{-\mu }C_{\mu }\frac{\pi \;\left(\omega_{\mu }+\omega_{-\mu }\right)}{8Q_{l}}\frac{1}{1+\frac{4Q_{l}^{2}{\left(D_{2}\mu \right)}^{2}}{{\left(\omega_{\mu }+\omega_{-\mu }\right)}^{2}}}.$$

Specifically, dispersion induces a mismatch of the resonances from the zero‐dispersion case. This mismatch affects the phase‐matching condition for the SFWM process [37]. However, we note that the phase mismatch in the nonlinear process can be omitted, as the linewidth of the resonator is typically much narrower (order of 100 MHz) than the generation bandwidth of the nonlinear process (several THz). Thus, the phase mismatch can be approximated to be constant within one signal idler mode pair, which only influences the generation efficiency, but has no impact on the optimum ring to bus coupling of the MRR.

From [12], the optimum pair generation rate for the dispersion‐less case can be found at $\frac{3}{4}Q_{e}=Q_{i}$, which can be recovered from Equation A7, by setting $D_{2}=0$ and simplifying $\omega_{\mu }\approx \omega_{0}$. Experimentally, the loaded Q‐factor is readily accessible. The maximum pair generation occurs for a loaded Q‐factor; $Q_{l,\max}=\frac{6}{7}Q_{l,\text{crit}}$, where $Q_{l,\text{crit}}$ is the loaded Q‐factor under critical coupling.
